# A Case Report of *Mycoplasma pneumoniae*-induced fulminant myocarditis in a 15-year-old male leading to cardiogenic shock and electrical storm

**DOI:** 10.3389/fcvm.2024.1347885

**Published:** 2024-04-16

**Authors:** Chengrui Zhu, Bo Hu, Xiaotong Li, Weiying Han, Yingjian Liang, Xiaochun Ma

**Affiliations:** Department of Critical Care Medicine, The First Hospital of China Medical University, Shenyang, Liaoning, China

**Keywords:** *Mycoplasma pneumoniae*, myocarditis, cardiogenic shock, ventricular arrhythmia, case report

## Abstract

*Mycoplasma pneumoniae (M. pneumoniae)* is a well-recognized pathogen primarily associated with respiratory tract infections. However, in rare instances, it can lead to extrapulmonary manifestations, including myocarditis. We present a case of a 15-year-old male who developed fulminant myocarditis, cardiogenic shock, and cardiac electrical storm attributed to *M. pneumoniae* infection. He underwent a combination of intra-aortic balloon pump (IABP) and veno-arterial extracorporeal membrane oxygenation (VA-ECMO) for cardiac support, ultimately surviving despite the intracardiac thrombus formation and embolic stroke. Following comprehensive treatment and rehabilitation, he was discharged in stable condition. This case underscores the importance of considering atypical pathogens as potential etiological factors in patients presenting with cardiac complications, especially in the adolescents. It also emphasizes the need for clinical vigilance and effective support for potential cardiac complications arising from *M. pneumoniae* infection.

## 1 Introduction

*Mycoplasma pneumoniae* (*M. pneumoniae*) is one of the main pathogens of community-acquired pneumonia (CAP), accounting for 10%–40% of such instances, with children and young adults as the most susceptible group (1, 2). In the year of 2023, following the cessation of COVID-19 restrictions, there were increasing patients admitted to hospital due to *M.pneumoniae* infection in China (3). Typically, *M. pneumoniae* causes mild respiration infection, however, some patients with *M. pneumoniae* may develop severe respiratory failure requiring ICU admission (4, 5). Moreover, *M. pneumoniae* can also manifest with extrapulmonary illness, including gastrointestinal, cardiovascular, neurological, renal, musculoskeletal, skin, and hematologic syndromes (1). These complications have been reported to occur in approximately 25% of individuals infected with *M. pneumoniae* and may occur at any time during the infection (6).

The cardiac manifestations of *M. pneumoniae* are rare. The cardiac manifestations of *M. pneumoniae*, including myocarditis, pericarditis, and conduction abnormalities, occurring in only 1%–8.5% of cases. Notably, this phenomenon is more prevalent in adults than in children (6). While relatively uncommon, these cardiac complications potentially can lead to severe consequences. Moreover, *M. pneumoniae*-induced fulminant myocarditis is an exceptionally rare occurrence, with no pertinent data reported to date. Herein, we present an unusual case of *M. pneumoniae* infection presenting with CAP, acute fulminant myocarditis, leading to cardiogenic shock, electrical storm, along with left ventricular thrombi and cerebral embolism, who survived with additional cardiac support with veno-arterial extracorporeal membrane oxygenation (VA-ECMO) and intra-aortic balloon pump (IABP) in a 15-year-old male.

## 2 Case description

A previously healthy 15-year-old male with no prior medical history was admitted to the emergency room (ER) in July 2023, after experiencing 8 days of fever, reaching temperatures as high as 40°C, accompanied by a dry cough. He had previously received intravenous azithromycin and cephalosporin antibiotics in the local clinic. Three days prior to admission to the ER, he developed dyspnea and coughed with white and pink foam sputum.

**Figure 1:**
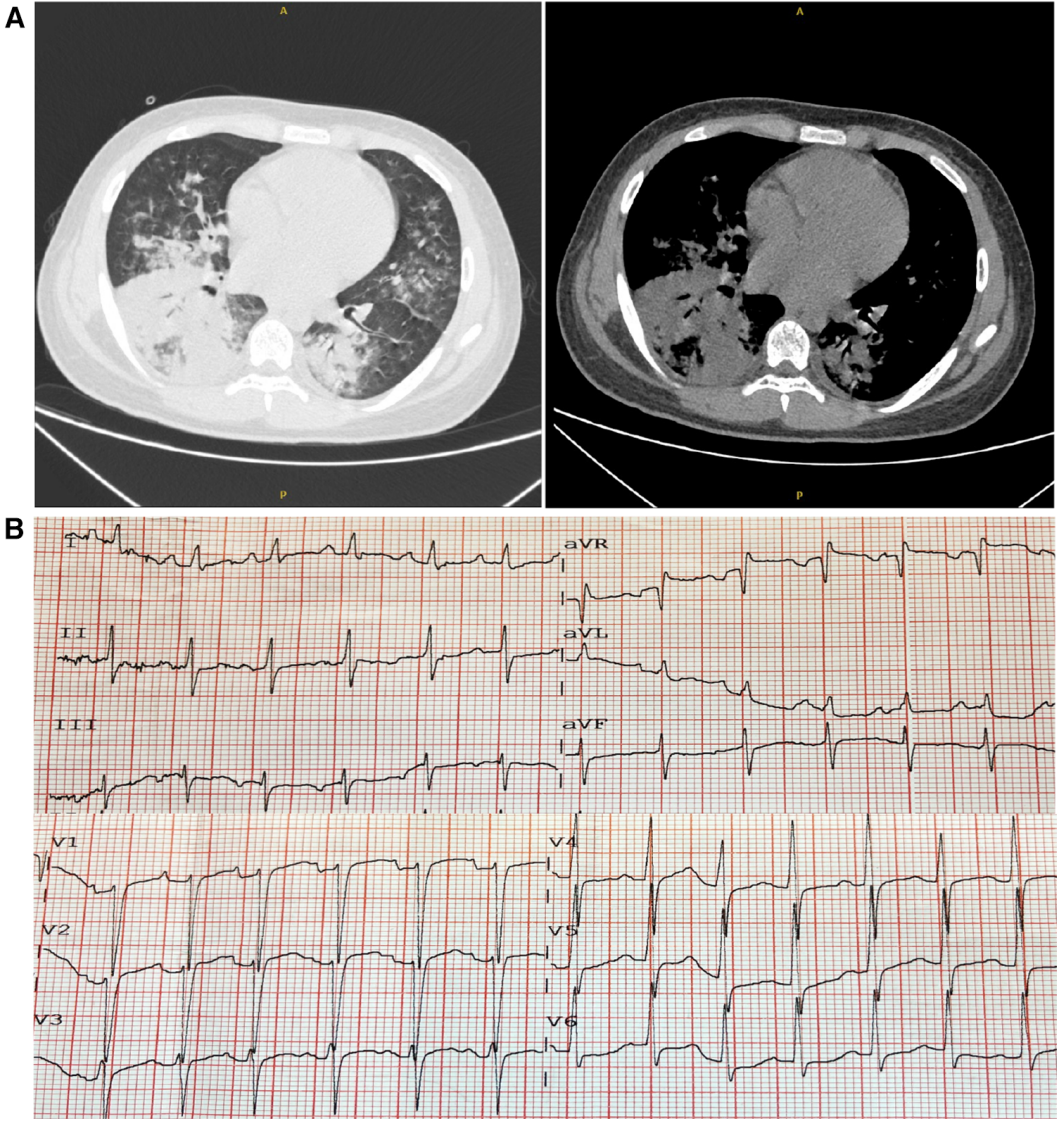
Chest computed tomography (CT) and electrocardiogram (ECG) on admission. (**A**) The chest CT scans revealed consolidation and patchy infiltrates within the right lower lobe and the left lower lobe. (**B**) The ECG showed sinus tachycardia accompanied by ST-T alterations.

On arrival at the ER, his vitals included a temperature of 39.5°C, heart rate of 153 beats per minute, respiratory rate of 35 breaths per minute, oxygen saturation (SpO_2_) of 90% on room air, and blood pressure of 97/63 mmHg. His weight was 60 kg, and his height was 175 cm. The chest computed tomography (CT) scan images on admission indicated consolidation of the right lower lobe and patchy infiltrates at the left lower lobe (Figure 1A), and the ECG showed sinus tachycardia and ST-T changes (Figure 1B). In addition, echocardiography revealed poor left ventricular systolic function with global hypokinesia [ejection fraction (EF) 30%]. Laboratory findings showed an elevated troponin-T and natriuretic peptide (BNP). He was diagnosed with CAP and acute myocarditis, receiving supportive care. Alongside, piperacillin/tazobactam, as empirical antibiotics, and 40 mg methylprednisolone were administered. The next day, he was transferred to the intensive care unit (ICU) for further support.

**Figure 2:**
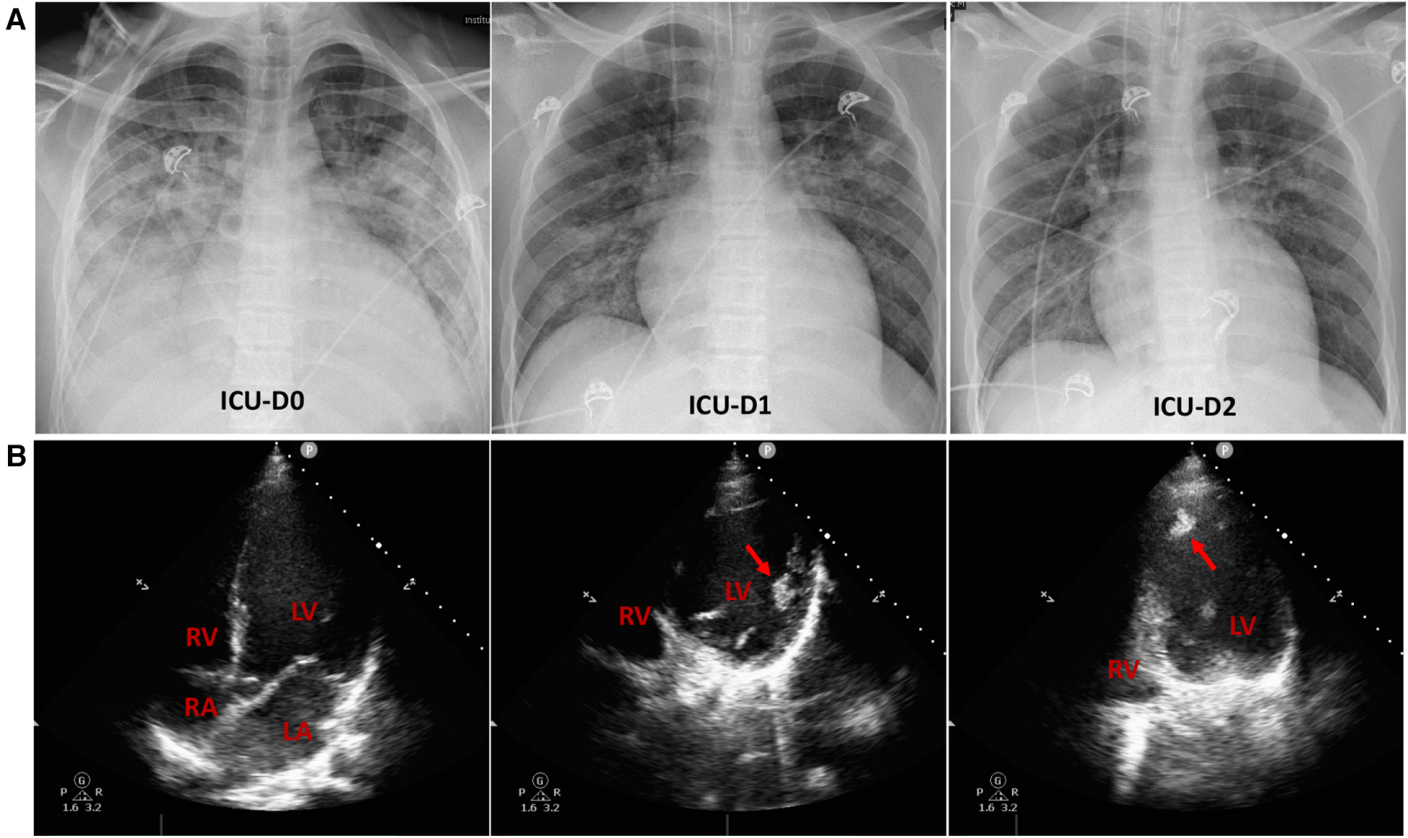
Chest radiographs and echocardiograms in the intensive care unit (ICU). (**A**) Chest radiographs on the initial three days of ICU admission. Lung edema exhibited a rapid amelioration. (**B**) On ICU admission, the apical 4-chamber views of the echocardiography indicated enlarged left atrium and ventricle, along with apical thrombi (indicated by red arrows) in the dilated left ventricle. RV, right ventricle; RA, right atrium; LV, left ventricle; LA, left atrium.

Upon admission to the ICU, the patient presented with a high fever, reaching up to 38.9°C. He received high-flow nasal oxygen (HFNO) therapy (60 L/min, FiO_2_ 50%), resulting in an SpO_2_ of 100%. To alleviate cardiac preload and pulmonary edema (Figure 2A), non-invasive ventilation was intermittently employed. The patient's blood pressure remained within normal range on low-dose vasopressors (metaraminol at 0.75 ug/kg*min), while his heart rate ranged from 130 to 150 beats per minute. Urine output and lactate levels were within normal limits, although the extremities exhibited signs of coldness. Low-dose inotropes, diuretics, beta-blockers, morphine, natriuretic peptide, and anticoagulants were administrated. The extra-cardiac organ functions were almost normal (Table 1).

**Table 1:** Clinical and laboratory data during the first 2 weeks of hospitalization.

Parameters	ER	ICU-D0	ICU-D1	ICU-D2	ICU-D3	ICU-D4	ICU-D5	ICU-D7	ICU-D9	ICU-D12	ICU-D14
Main intervention				CPR + IABP + IMV		ECMO				Withdrawal of ECMO	
T, °C	39.5	38.9	38.7	37	38.4	37.8	36.8	37.4	37.3	37.3	37.2
HR, bpm	153	151	90	108	73	78	80	85	94	82	76
Vasopressors, ug/kg*min	DA 3	MA 0.75	MA 0.5	NE 2	NE 1.4	NE 1.6	NE 1.0	NE 0.4	NE 0.4	NE 0.2	NE 0.2
Urine output, ml		12 h: 600	3,910	3,605	2,600	5,320	5,570	3,120	3,055	1,780	2,420
Lactate, mmol/L	2.2	1.6	1.4	2–15	2.3–5.5	2.6	3.2	2.3	1.9	1.1	2
WBC,10^9^/L	11.49	14.42	13.61	10.17	18.92	20.16	19.87	13.68	27.19	15.46	12.19
Ly, 10^9^/L	1.35	1.25	1.66	2.23	4.43	2.53	0.87	0.84	4.82	3.83	3.18
PLT,10^9^/L	211	228	280	264	314	195	147	130	93	57	110
PT, s	14.3	15.6	14.8	14.4	14.3	16.3	15.7	15.7	15.7	17.7	17.2
APTT, s	38.7	36.8	39.6	43.2	39.6	47.1	58.4	92.5	159.9	161	81.7
Fib, g/L	5.12	5.94	5.47	5.29	4.31	4.19	3.55	2.57	1.75	1.54	1.6
D-dimer, ug/ml	2.57	9.58	9.57	9.81	7.26	2.19	2.87	2.43	3.01	10.65	5.72
ALT, U/L	93	97	87	84	94	79	105	106	92	112	95
T-bil, μmol/L	7	9.5	9.2	9.4	9.2	12.4	20	22.5	20.9	14.4	23.9
Cr, μmol/L	69	62	57	59	107	104	81	61	53	51	48
CRP, mg/L	77.35		116.2		55.8				23.5		22
TnI, ng/ml	TnT 0.334	3.983	3.385	1.96–6.37	4.95–2.45	3.571	1.684	0.599	0.345	0.096	0.049
NT-proBNP, pg/ml	4,421	6,595	11,670	13,016	17,383	8,950	1,312		1,102		BNP 211
EF, %	30%	26%		20%		20%	22%			30%	

The values represent the worst values observed throughout the day.

ALT, alanine aminotransferase; APTT, activated partial thromboplastin time; BNP, b-type natriuretic peptide; CPR, cardiopulmonary resuscitation; Cr, creatinine; CRP, C-reactive protein; DA, dopamine; ECMO, extracorporeal membrane oxygenation; EF, ejection fraction; ER, emergency room; HR, heart rate; IABP, intra-aortic balloon pump; IMV, invasive mechanical ventilation; Ly, lymphocyte; T, body temperature; WBC, white blood cell; PLT, platelet; PT, prothrombin time; Fib, fibrinogen; T-bil, total bilirubin; TnI, troponin I; MA, metaraminol; NE, norepinephrine.

Cefoperazone/sulbactam and azithromycin were empirically started to cover the potential pathogens. Simultaneously, nasopharyngeal swabs and plasma samples were screened for pathogens associated with myocarditis and CAP. Additionally, blood and sputum samples were sent for metagenomic Next Generation Sequencing (NGS) assay (ID seq™ Ultra, Vision Medicals Co., Ltd., Guangzhou, China) to identify the causative pathogens. These results revealed a significant increase in *M. pneumoniae* antibodies [IgG > 300 AU/ml detected by chemiluminescence immunoassay commercial kits (YHLO Biotech Co., Ltd., Shenzhen, China)] and the presence of *M. pneumoniae* in both blood and sputum in two days, confirming the diagnosis of *M. pneumoniae* infection. Furthermore, in response to the positive result of *M. pneumoniae* infection, sputum sample was sent to a targeted NGS assay (KingMad Co., Ltd., Guanzhou, China) to detect macrolide-resistant genes, revealing a point mutation of A2063G on the 23S rRNA (12 reads out of 341 reads of *M. pneumoniae*). Consequently, tigecycline was administered in combination with azithromycin on ICU-day 2, as quinolones are contraindicated for patients under 18 years of age. A dosage of 40 mg/day methylprednisolone was administered during the initial five days in ICU.

The echocardiography conducted in ICU indicated prominently decreased left ventricle contractility with an EF of 26%, enlarged left atrium and ventricle [Left ventricular end-diastolic volume (LVEDV) 223 ml], along with apical thrombi in the dilated left ventricle (Figure 2B**)**. Following that, the anticoagulant therapy was strengthened with subcutaneous injection of 4,100 IU of low molecular weight heparin (LMWH) every 12 h.

With the initial support, both lung edema and hemodynamic condition showed rapid improvement (Figure 2A, Table 1). However, at noon on the third day of ICU admission, he experienced an episode of ventricular fibrillation (VF) and Adams–Stokes syndrome. Cardiopulmonary resuscitation (CPR), defibrillation, endotracheal intubation, and brain protection were initiated, and amiodarone was infused. Spontaneous circulation was restored 20 min later under advanced cardiac life support. Nevertheless, after resuscitation, his condition significantly deteriorated, with aggravating cardiogenic shock and the presence of severe pulmonary edema. In response, IABP was immediately administered with a counter pulsation ratio of 1:1, and a counter pulsation pressure of 120 mmHg. With the IABP support, peripheral circulatory perfusion was improved, and the pulmonary edema declined. He experienced intermittent ventricular premature beats, which could be reversed by antiarrhythmic drugs. However, on the subsequent day, he experienced refractory ventricular tachycardia (VT) and VF. Unfortunately, these episodes constituted an electrical storm (ES) that could not be successfully terminated despite attempts with defibrillation, CPR, and antiarrhythmic drugs. Consequently, VA-ECMO was initiated through the femoral artery and vein cannulation, with an initial flow set at 3 L/min. To prevent distal leg ischemia, a catheter connected superficial femoral artery with femoral artery was employed. The combined support of VA-ECMO and IABP, allowed for aortic valve opening during left ventricular contraction.

After two days of ECMO initiation, he no longer exhibited malignant arrhythmia. Meanwhile, his cardiac enzyme and BNP gradually declined (Table 1). Hemodynamic stability was achieved with NE at 0.2–0.4 ug/kg*min, and daily bedside echocardiography demonstrated gradual improvement in cardiac contractility. Accordingly, the ECMO flow rate was decreased, according to the vital signs and indicators of peripheral circulatory failure. Concurrently, pharmacotherapy for heart failure, including low doses of angiotensin-converting enzyme inhibitors and beta-blockers, were administered to mitigate the risk of ventricular remodeling.

The clinical course and laboratory data during the initial 14 days in ICU were outlined in Table 1. The chest images revealed a significant resolution of pneumonia. On ICU Day 12, he was successfully weaned from ECMO, when echocardiography showed an estimated LVEF of 30% and LVEDV of 220 ml. The IABP was subsequently removed on ICU Day 16. Nevertheless, the thrombi in the left atrium persisted. Throughout the period of mechanical circulatory and ventilation support, spontaneous awakening trials were implemented, and the patient remained conscious despite prior episodes of CPR.

**Figure 3:**
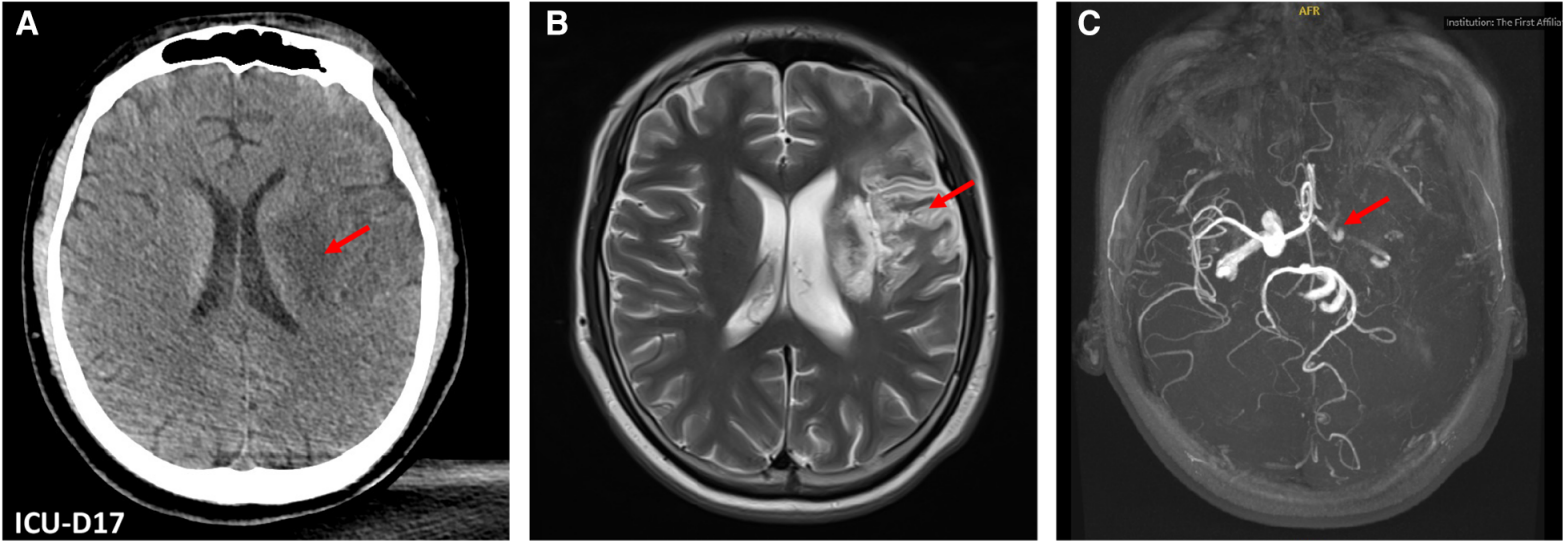
Brain computed tomography (CT) and magnetic resonance (MR) images. (**A**) Brain CT on ICU Day 17 revealed an acute infarction of a large area of left middle cerebral artery territory (red arrow). (**B**) The post-ICU discharge brain MRI showed hyperintense regions (red arrow) in the left frontotemporal and basal ganglia areas. (**C**) Brain MR angiography revealed diminished perfusion in the intracranial segment of the left cervical internal carotid artery left, along with occlusion of the left middle cerebral artery (red arrow).

Unfortunately, he experienced an acute ischemic stroke resulting in right-sided hemiplegia on the 17th day of ICU stay. Brain CT revealed an acute infarction of a large area of left middle cerebral artery territory, consistent with an embolic pattern. This was likely attributed to the dislodgement of the left ventricular thrombus, a finding subsequently confirmed by magnetic resonance imaging and magnetic resonance angiography after discharged from ICU (Figure 3). The anticoagulant therapy with LMWH was continued, although thrombolysis was not administered due to the risk of secondary bleeding and a probable prolonged duration of ischemic stroke (exceeding 6 h). Furthermore, serial CT scans were performed over two weeks to monitor for potential hemorrhagic transformation.

Following continued stabilization of cardiac function and active physical rehabilitation, he was successfully weaned from mechanical ventilation on the 20th day of his ICU stay. The following bedside echocardiography demonstrated a gradual resolution and eventual disappearance of the intracardiac thrombi. Throughout the subsequent hospitalization period, there were no further complications or reports cardiac discomfort symptoms. He was ultimately discharged from the hospital on the 37th day of admission and returned home for further physical rehabilitation.

Three months after discharge, he remained free from any cardiac discomforts. However, a follow-up echocardiography persistent revealed left ventricular enlargement, albeit with a reduced LVEDV of 149 ml, and an increased EF of 42%, compared to previous measurements. Through diligent limb rehabilitation exercises, he demonstrated the ability to stand and walk independently.

## 3 Discussion

In this report, we present a complex and unusual case of *M. pneumoniae* infection characterized by the occurrence of fulminant myocarditis, cardiogenic shock, malignant ventricular arrhythmia leading to electrical storm, along with left ventricular thrombosis and subsequent embolic stroke in a healthy 15-year-old male.

Viruses are the predominant pathogens among infectious causes of myocarditis, while mycoplasma infection-induced myocarditis constitutes a minor proportion of cases. Typically, *M. pneumoniae* causes mild respiration infections with potential extra-pulmonary manifestations. However, increased clinical severity and extrapulmonary manifestations have been reported to be associated with delayed effective treatment for macrolide-resistant *M. pneumoniae* (7, 8). In this case, a low sequence number of *M. pneumoniae* with a macrolide-resistant gene, A2063G on the 23S rRNA, was detected. A2063G mutation can decrease the affinity of macrolide with *M. pneumoniae* ribosome, accounting for 99% of macrolide-resistant *M. pneumoniae* infections in China (9). The existence of macrolide-resistance in *M. pneumoniae* might partially contribute to the fulminant cardiac complications in the patients. Tigecycline was subsequently administered in combination with azithromycin, targeting the potential macrolide-resistant strains. However, due to the limited sequences of the macrolide-resistant *M. pneumoniae*, a definitive causal relationship between the fulminant clinical course and macrolide resistance cannot be established.

The *M. pneumoniae*-induced myocarditis can lead to severe consequences, as demonstrated in this case. The precise mechanisms underlying *M. pneumoniae*-induced myocarditis are multifaceted and not yet entirely elucidated. It involves the activation of the host immune response, leading to release of pro-inflammatory cytokines, such as interleukin-6 and tumor necrosis factor-α, within the cardiac tissues (6, 10). Furthermore, *M. pneumoniae*-induced myocarditis may involve autoimmune mechanisms. The cross-reaction between the *M. pneumoniae* cell components and cardiac tissues plays a role in the cardiac injury (10). Increased levels of circulating immune complexes and elevated T-cell immunoglobulin and mucin domain 1 titers have been associated with myocardial damage in *M. pneumoniae* infections (11, 12), highlighting their significance in the development of *M. pneumoniae*-induced myocarditis. Additionally, it has been suggested that *M. pneumoniae* may directly invade cardiac cells, causing cellular damage and further contributing to the inflammatory cascade (13).

While the *M. pneumoniae*-induced CAP in this case was promptly resolved with the appropriate antibiotics and supportive care, the myocarditis and the severe cardiac presentation necessitated ICU admission and advanced cardiac support measures. During the initial two days in ICU, cardiac outputs and perfusions could be maintained with low dose of vasopressors and inotropes. However, as the patient developed ventricular arrhythmia and ES, the hemodynamic status of the patient rapidly deteriorated. ES, characterized by three or more separate episodes of ventricular arrhythmia within a 24-hour period, poses a high risk of sudden cardiac death, significant reduction in quality of life, and an overall poor prognosis (14). The ES observed in this case could be associated with the unstable cardiac electrical activity triggered by the myocardial inflammation, along with the altered electrophysiological property due to ventricular remodeling and dilation (14, 15).

Due to inadequate efficacy of pharmacological interventions, mechanical circulatory support was necessary to ensure systemic and coronary perfusion, facilitate venous drainage, and prevent multiple organ dysfunction (16). The timely application of IABP immediately following the initial episode of VF was chosen for its convenience and cost-effectiveness. By employing counterpulsation, IABP reduce left ventricular afterload and increase forward blood flow to the brain and kidney, providing approximately 20% additional circulation support (17). Indeed, this intervention substantially improved perfusion under conditions of declining cardiac output. Unfortunately, the occurrence of ES made it unable to optimize balloon inflation/deflation, resulting in inadequate perfusion. Hence, VA-ECMO were considered immediately under this circumstance. VA-ECMO, recognized as the most advanced temporary circulatory support, ensures immediate and comprehensive hemodynamic support (18). VA-ECMO decreases cardiac preload, improves systemic perfusion, reduces myocardial oxygen consumption; however, it can potentially lead to left ventricular overload and distention due to the increased afterload (18). Therefore, VA-ECMO is better employed in combination with IABP, and in a setting of low flow rate, to effectively mitigate afterload and maintain pulsatile flow. In this case, the combined application of VA-ECMO and IABP played a pivotal role in stabilizing the patient's hemodynamics, allowing for cardiac recovery and, ultimately, weaning from the circulatory supports.

In previous reports, cardiac thrombosis associated with *M. pneumonia* is extremely rare (19, 20). In the case presented, the persistence of left ventricular thrombi posed a significant challenge in clinical management. The development of left ventricular thrombi could largely be attributed to intracardiac turbulence resulting from the ventricular enlargement and the compromised systolic function. Furthermore, *M. pneumoniae* infection also leads to vascular occlusion a which may lead to subsequent cardiac thrombosis. This occlusion can result from either localized vascular injury, or a systemic hypercoagulable state mediated by cytokines (10, 21). Despite anticoagulant therapy was administered initially, the risk of embolic events persisted, ultimately leading to an acute ischemic stroke resulting in right-sided hemiplegia. This underscores the importance of anticoagulant management and vigilance for thromboembolic events and in patients with myocarditis caused by *M. pneumonia*. Management strategies for preventing coagulopathy and thromboembolic events in this case included anticoagulant therapy and monitoring to reduce thrombus propagation and the risk of recurrent events or bleeding complications. Additionally, monitoring of the left ventricular thrombus and heart failure management were employed to prevent further embolization. In some cases, thrombolysis and intracardiac thrombectomy may be considered; however, thrombolysis was not administered due to the risk of secondary bleeding and the likelihood of a prolonged duration of ischemic stroke. Gradual resolution and eventual disappearance of the intracardiac thrombi were observed, indicating a positive therapeutic response without the need for invasive procedures.

Complete cardiac recovery is common in myocarditis associated with *M. pneumonia* infection; however, long-term sequelae have been described (22, 23). In the presenting case, while the patient's cardiac recovery has been encouraging, there was persistent left ventricular enlargement three months after discharge, which could potentially lead to further cardiac complications, such as dilated cardiomyopathy, heart failure, arrhythmias, and sudden cardiac death. This discovery prompts inquiries into the long-term cardiac consequences of *M. pneumoniae*-induced myocarditis, suggesting the need for ongoing cardiological follow-up.

## 4 Conclusions

In conclusion, this case illustrates the diverse clinical manifestations of *M. pneumoniae* infection, demonstrating its potential to trigger fulminant myocarditis leading to cardiogenic shock, electrical storm, as well as the formation of left ventricular thrombi and cerebral embolism. It underscores the importance of maintaining clinical vigilance for potential cardiac complications associated with *M. pneumoniae* infection. Additionally, it emphasizes the necessity for prompt implementation of advanced cardiac support measures to ensure positive outcomes.

## Data Availability

The raw data supporting the conclusions of this article will be made available by the authors, without undue reservation.
